# Electricity a Paralyzing Agent

**Published:** 1879-11

**Authors:** Thos. W. Poole


### ELECTRICITY A PARALYZING AGENT.

BY THOS. W. POOLE, M. D., M. C. P. AND S. ONT.

Among the standard writers on medical electricty this agent is regarded as an "alterative," a "tonic," and a "stimulant;" while by some it is said to possess these qualities in combination. Experimental physiologists invariably regard it as an "excitant," or "irritant" to nerve-tissue, and interpret the effects produced in accordance with this view of its action. It may be thought very bold in us to call in question opinions so generally received; but as we are persuaded the view of the action of electricity we are about to present is well founded, and especially as we shall appeal to the recognized authorities themselves in support of our thesis, we ask for it a fair and candid hearing.

We assert that electricity is a paralyzer of nerve-tissue, and we claim that all the effects it produces, as employed for therapeutical and physiological purposes, may be readily accounted for on this view of its action. In proof of its paralyzing effects on nerve-tissue, we point, first, to Matteucci's experiment on the spinal cord of a living rabbit, in which it was shown that during the passage of a common galvanic current the cord might be pricked, cut, torn, burned, or otherwise injured, without eliciting from the animal any sign of pain. Dr. C. B. Radcliffe, F. R. S., quotes this experiment (with others) in his "Lectures on Epilepsy, Paresis, and Pain," and there distinctly asserts "the paralyzing influence" of electricity: "Whether the current was passed up the spine or down the spine, the result was the same so far as *its paralyzing action* was concerned, and so it was in the other two experiments which have been mentioned" (p. 65). Dr. Radcliffe, however, asserts that the muscles, as well as the nerves, are paralyzed by the galvanic current, to which, as well as to some other of his conclusions, we take exception, for reasons which will hereafter appear. Drs. Beard and Rockwell, leading medical electricians of New York, also testify to the same effects in stating that "the cord remains insensible to any stimulus that may be applied to it, so long as the current is passing." (Med. and Surg. Elec., 2d ed., p. 127). Dr. Moritz Meyer refers to the observations of Valentine, Matteucci, and Eckhard in this connection, and to the effects of the constant current on nerve-tissue, and adds: "In other words, *the nerve is paralyzed* so long as any portion of it is subjected to the action of a continuous current" (Elec. in Prac. Med. Hammond, p. 62).

The faradic current produces similar paralyzing effects, which differ from those of the galvanic current "mainly in degree." Thus the faradic current has been effectually employed to benumb local sensation in parts about to be subjected to minor surgical operations, and, as is well known, both currents are employed for the temporary relief of neuralgia and other painful states; for which result no better explanation can be offered than that the nerves of the affected part are for the time so paralyzed as to render them incapable of transmitting the sensation of pain. It may be said that this effect is properly anæsthesia rather than paralysis; but these are only different names for the same condition, and as Dr. Anstie has fully shown, anæs hestics are invariably paralyzers (Stim. and Narcot., pp. 273, 328, 398, etc.) Electricians frequently allude to "the benumbing effects" of electricity, including both the galvanic and faradic currents (Drs. Beard and Rockwell, loc cit., pp, 123). When the function activity of a nerve is not simply perverted, but arrested, it may with propriety be said to be paralyzed, and the agent producing this effect is certainly entitled to be called a stimulant, or its action that of an "excitant," as has been so much the rule in regard to electricity.

It is important here to note, as negative evidence of the truth of our proposition, that Prof. Trowbridge, of Harvard College, U. S., has demonstrated that the supposed natural currents of electricity, believed by Matteucci and Du Bois-Reymond to be present in nerve and muscle in repose, do not exist; but that the perturbations of the index of the galvanometer witnessed by them really depended upon currents developed in the galvanic accessories of the experiment, and not in the nerves or muscles themselves; so that, so far as known, electricity plays no part whatever in the inter-relations of nerve and muscle. Nor is electricity known to be generated at all in the bodies of men and animals, since even in the case of certain electric fishes, which display a remarkable use of this agent, the electricity which they discharge may be simply *induced* in them by the opposite electricity of the media surrounding them, as appears to be probably the case in certain men and women who give out sparks of electricity —condition largely dependent on the electric state of the atmosphere, that of their surroundings, and their own peculiar electric relations to these. It is true that electricity is found *on the surface* of animal and other bodies, in the static form, in which, so far as is known, it is inoperative and exerts no influence on vital processes, as the negative result of "charging" the surface of the body with electricity on an insulated stool serves as an illustration. In view of the present state of knowledge on this subject, those who assert that natural electricity plays any part in the vital operations of the living organism will have to begin anew the task of eliciting evidence in support of that doctrine.

Turning now again to the subject of artificial electricity, the question at once arises, If this agent be a paralyzer of nerve-tissue, how does it "stimulate" the muscles to contract in the manner so commonly witnessed? The reply to this inquiry involves an issue which we have attempted to discuss at length, in a recently published work, entitled "Physiological Thereapeutics," and at which we can only glance in the space available here.

We are convinced that the nerves do not "stimulate" the muscles to contract. We find all the physiologists asserting the inherent contractile power of muscular fibre, and even admitting the ability of muscle to exercise this power, in many instances, quite independently of nervous influence. Illustrations of this are to be found in the movements of microscopic portions of protoplasm; in the contractions of the fœtal heart before the development of nerves; in rythmical and other movements of muscular parts after death of the body; in post-mortem parturition in pregnant females who had died undelivered at or near the end of the period of gestation; in parturition normally occurring in cases where the spinal cord had been destroyed by disease, or paralyzed previously, well authenticated case of which are mentioned by Dr. W. B. Carpenter (Human Physiology, 5th Am. ed., pp. 979, 980); in the existence of rigor mortis, the facts of which have not been explained on any satisfactory basis (not excepting the latest hypothesis, which refers it to coagulation of the myosin or muscle plasma), other than that of independent muscular contraction, which is the view of the late Dr. Anstie and of physiologists generally.

Dr. C. B. Radcliffe, in the work already quoted, asserts (in support of a different thesis) that "muscles do not pass into a state of contraction when they may be supposed to receive a larger supply of nervous influence than usual;" and, again, that "ordinary muscular contraction is associated with deprivation of nervous influence;" nay, more, "that *the power of muscular contraction is inversely related to the amount of nervous influence supplied to the muscles from the nervous centres*" (pp. 95–100). The experiments of Sir A. Cooper, Kussmaul and Tenner, and Dr. Brown-Sequard, are quoted in support of these propositions, which find additional confirmation in such facts as the cure of a much larger percentage of cases of tetanus by stimulants than by any other means (Dr. W. A. Hammond, Diseases of Nerv. Syst., 540). It is only on the view that in the excessive muscular contraction here witnessed nerve-force was in abeyance (and not in excess) that stimulants could be justifiable, or this result explained. Dr. Anstie was so impressed with the force of Dr. Radcliffe's argument that after referring to it he writes: "The true action of vital force would appear to be rather that of restraining muscular contraction than of exciting it," (Stim. and Narcot., p. 70).

Although Dr. Anstie does not appear to have further supported the opinion just quoted, it is certainly the view of the relations of nerve to muscle most consistent with the facts. It would really appear that whatever cause paralyzes nerve-force sets the muscle free to assert its inherent contractile power, and it does this with clonic or tonic results in proportion to its freedom.

Electricity paralyzes the motor nerves, and so permits muscular contraction. This principle, like a master-key, unlocks the fastnesses and clears up the obscure and hitherto unexplained problems in electrical treatment. It accounts for the benefit this agent sometimes affords to muscles suffering from enforced disuse, by the exercise it gives them, thus attracting blood, and with this pabulum, whereby their nutrition is improved. It accounts for the failure of electricity in chorea (Dr. J. Russell Reynolds, Clin. Uses, etc., p. 83) and in spasmodic states generally. Because the preponderance of muscular action here is owing to the too feeble restraint already exercised by weak or exhausted nerves, and an additional paralyzing agent is the least likely means to benefit them. It explains why electricity is powerless for good whenever, as in early and late rigidity, it fails to induce muscular contraction; because the muscle is already isolated from the nervous centre; the nerve is paralyzed and electricity can free it no further it fails to cause it to contract, and its use can do no good, but may do much harm. It also serves to explain why it is, "if you find, for instance, a limb perfectly paralyzed, but contracting perfectly well to galvanism, or sometimes acting even in excess, you can do nothing more by applying galvanism to that limb" (Dr. J. Russell Reynolds, Clin. Uses of Elec., p. 94). The benefits of electricity are for the muscle, in the manner already stated, but here the muscles are healthy and their condition cannot be improved. It is the nerve that is at fault, and electricity not being a tonic, or stimulant, or vitalizer to it, does not improve its condition, as it ought to do if it possessed the qualities attributed to it. Here is a problem: "Why the muscles that are paralyzed should act more readily than healthy muscles to a slowly interrupted current has not yet been explained" (ib., p. 98). The explanation is easy. A weaker current serves more to paralyze the nerves of the diseased limb, and set the muscles free to contract, than will suffice for the healthy nerves of the sound limb. Why a slowly interrupted galvanic current is sometimes effective for this purpose, where the faradic current fails, we have discussed elsewhere in our "Physiological Therapeutics," where the more important objections to the theory here advocated have also been considered.

Numerous facts might be adduced to show that the relations here assigned to motor nerve and muscle, apply also to the vaso-motor nerve and muscular coats of the arterioles But as this part of the subject is only indirectly associated with the topic of this paper, and as we have discussed it in a recent issue of the *Record*, under the title of "The Effect of Pithing on the Vascular System," we make no further reference to it here.

We have already shown, how, as a paralyzing agent, electricity indirectly benefits muscles whose functional power is impaired from atrophy or disuse—by improving their nutrition. A wide field of usefulness is also open to this agent—on the same view of its action as a nerve-paralyzer—in the control it exercises over vascular activity in organs and tissues. By paralyzing the vaso-motor nerves (whose function we claim to be to *dilate* the arterioles) it brings into play the independent contractile power of the muscular tissue of the arterial coats, and thus produces a reduction in the calibre of these vessels, arresting congestion and diminishing blood-supply to morbid growths or hypertrophied tissues, with the gratifying results not unfrequently recorded. Thus the undoubted beneficial results of electricity in certain cases, as well as its failure in others, find a ready explanation in the theory we advocate.

The present physiological theory of the action of electricity in arresting the heart is of a most extraordinary character. Faradization of the sinus venosus of the heart of the frog, at its junction with the auricle, brings the heart to a standstill. In order to account for the arrest of the heart by a reputed "stimulus," it was necessary to assume that it is a hypothetical "inhibitory" ganglion, which is thus excited so as to overpower the motor ganglia of the heart, and cause it to stop, and at the same time that the proper motor ganglia of the heart escape the "excitation" of the faradic current altogether.

Now, the chief motor ganglion of the heart of the frog is said by physiologists to be situated in the sinus referred to, close to the auricle, at the very point to which they apply the faradic current to arrest the heart. Why the motor ganglion, to which the current is thus directly applied, should be regarded as uninfluenced, while another ganglion, a little farther off in the septum, should be "excited" (though less directly in the range of the current), so as to master its reputed rivals and stop the heart, is a problem which requires explanation. We claim that the application of a faradic current to the chief motor ganglion of the heart paralyzes it directly, and so stops the heart; and further, in a paper in recent issues of the Canada *Lancet*, we have undertaken to show on physiological authority that the so-called "inhibitory," "accelerator," and "depressor" nerves of the heart do not discharge the functions these names imply; that they do not directly influence the heart at all, and so far as they influence it directly, they do so through "the peripheral circulation" in the manner of ordinary vaso-motor nerves.

We desire to refer briefly to the serious consequences which may, and have, resulted in practice from the mistaken idea that electricity is a tonic or stimulant. We refer especially to its use as a supposed restorative in cases of suspended animation, as in apparent drowning, or in threatened death from chloroform. The cases on record where apparent benefit has resulted from electricity in these states, are so few, so associated with other remedial processes, and generally so unsatisfactory, as to furnish no trustworthy evidence in its favor, while that to the contrary is direct and convincing. It has notably failed in experiments where these states were artificially produced to test its powers, in the hands of such experienced electricians as Drs. Beard and Rockwell (Med. and Surg. Elec., 2d ed., pp. 665–6). It has extinguished the spark of life in cases of threatened death, which were happily recovering under other means; and many more such catastrophes would be on record, if duly reported, and if it were not that batteries are frequently not available on such occasions—or when so, are often out of order, and fail to act.

Dr. Ringer, in writing of the use of electricity in these cases, states that "some authorities are wholly opposed to its use, on the score of its influence to arrest a very feebly beating heart, and so diminishing any slight remaining chances of recovery" (Therapeutics, p. 292). Dr. B. W. Richardson, of London, England, writing of resuscitation from the narcotism of chloroform artificially induced, states: "I feel it too unreasonable to recommend galvanic action as a means of resuscitation. Galvanism is a two-edged sword. It might by accident, I may say, in some cases, restore respiration, but it would in this respect be inferior in principle to artificial respiration, and in the majority of cases *it would more effectually promote death than restore life*. . . . When used as it is commonly used, merely to excite prolonged contraction of muscles, it is not aimless merely, but positively mischievous." Having narcotized a rabbit with chloroform till respiration and other evidences of life had ceased, and restored it by artificial respiration, he narcotized another to a similar degree, to show the effects of electricity. Commenting on the fatal result, he states: "When I used the electric stimulus [observe the word employed in this connection], I took out of the muscles what remaining force was there—the primary force required for recovery—and under the semblance of restoring life, *clenched death!*" (*Medical*

*Times and Gazette*, 1870. Braith. Retros., January, 1871, p. 256.)

How well this tallies with the action of electricity as a paralyzer, and how very inconsistent it is with its action as a reputed stimulus! Yet such is the force of habit, custom, or blind adherence to authority, that men call that a stimulus while in the very act of recording its paralyzing effects. Nor is Dr. Richardson happy in his allusion to taking the force out of the muscles. The muscle is not paralyzed by electricity. It will soon pass into rigor mortis, in which it will display "the most steady and persistent contraction which muscle can possibly exhibit," to use the words of Dr. Anstie, a condition which electricity simply hastens. If Dr. R. had stated that by means of the electric current he had intensified the already existing paralysis of the nerves (produced by the chloroform), and thus prevented all chance of recovery, his statement would have been more in accord with his facts, and with the ideas he evidently intended to convey. Let us be courageous, and call a spade a spade. "A rose by any other name would smell as sweet," but it is highly dangerous to label certain paralyzers roses—or stimuli—and proceed to employ them accordingly.

This fatal error has warped the whole course of physiological research, and given rise to the most absurd and contradictory statements in therapeutics. As an example, we are told by Preyer that "belladonna paralyzes the peripheral branches of the vagus and at the same time stimulates the nervous centres of respiration."

The fundamental error regarding electricity is responsible for this statement of contrarieties. Here is the process by which such a conclusion is reached. Electricity is said to be a stimulus. But it stops the heart. Then it must be assumed to excite (not the heart's motor ganglia, which would urge it faster, but) a nervous rival antagonistic to the motor ganglia. Hence the assumption of the "inhibitory" mechanism, which so to say, is supposed to apply the brakes to the heart's *motor* ganglia. It follows from this, that to urge the heart to increased action we must stimulate its motor ganglia; to arrest it, we excite the inhibitory nerves. The action of drugs is interpreted according as they seemed to act in one or other of these ways. Belladona quickens the heart's action (under certain conditions.) Hence it is said to paralyze the inhibitory ganglia (Dr. Ott) and also to paralyze "the peripheral branches of the vagus," which are understood to be connected with the inhibitory centre in the heart. By another chain of reasoning it is concluded that it excites the vagus as its origin.

It is pertinent here to inquire how the heart's motor ganglia escape the effects of the drug which is assumed to paralyze their rival? They are in proximity, in the same organ, fed by the same blood-stream, and yet one is held to be uninfluenced while the other is paralyzed. Again, Dr. Carpenter states that the membranous sheath of nerve-fibres "is not penetrated by blood-vessels." How then can the "branches" of a nerve trunk be influenced by poisoned or medicated blood, except by impressions made on the nerve-centre and transmitted along the trunk? And if this be true physiology, how can a nerve-centre and its trunk, of "branches," be in opposite conditions of excitation or paralysis? Is it not preferable to believe with Dr. Anstie that the action of narcotics "is an uniform one, and tends entirely in the direction of nervous death?"

A recent physiological author writes of belladonna: "It paralyzes the motor nerves of frogs at the same time that it excites the spinal cord; after they recover from the motor paralysis, the tetanic symptoms of spinal stimulation appear." (Dr. Ott, Action of Drugs, p. 138. Is it by such teachings as this that we can ever learn the true action of drugs? Surely the hypothetical "Philadelphia lawyer," who is reputed able to solve all difficulties, has been at work here, preparing "a case" for the veritable Philadelphia doctor! But seriously, the inferences drawn from electrical experimentation, as to the functions of organs, when applied to the observed effects of drugs on these functions, have led even eminent writers into many such absurdities.

Electrical experimentation has borne a very prominent part in formulating the current views of the functions of certain parts of the organism, and has assisted in building up a very complex theory both of these functional activities and of the action of drugs in relation to them. A recognition of the true action of electricity as a paralyzer, instead of as a stimulus to nerve-tissue, would, we believe, be a great gain to both physiology and therapeutics.—*Medical Record.*

